# Dynamic Phenotypes and Molecular Mechanisms to Understand the Pathogenesis of Diabetic Nephropathy in Two Widely Used Animal Models of Type 2 Diabetes Mellitus

**DOI:** 10.3389/fcell.2020.00172

**Published:** 2020-03-19

**Authors:** Yanfei Liu, Hui Huang, Rui Gao, Yue Liu

**Affiliations:** ^1^Cardiovascular Diseases Center, Xiyuan Hospital of China Academy of Chinese Medical Sciences, Beijing, China; ^2^Graduate School, Beijing University of Chinese Medicine, Beijing, China; ^3^Institute of Clinical Pharmacology of Xiyuan Hospital, China Academy of Chinese Medical Sciences, Beijing, China; ^4^Beijing Duan-Dian Pharmaceutical Research & Development Co., Ltd., Beijing, China

**Keywords:** diabetic nephropathy, type 2 diabetes mellitus, pathogenesis, sprague dawley rat, KK-Ay mice

## Abstract

**Objective:**

We aimed to characterize the pathogenesis of diabetic nephropathy (DN) in two commonly used type 2 diabetes mellitus (T2DM) animal models and explore the preliminary molecular mechanisms underlying DN in two models.

**Methods:**

To verify the effect of hyperglycemia on renal tissue, we observed the cell growth inhibition rate by adding different concentration of glucose to cell supernatant. After that, a chemically-induced T2DM model was established by administering streptozotocin (STZ) to Sprague Dawley (SD) rats in combination with high fat feeding. In addition, a spontaneous T2DM model was established by feeding 8 weeks old KK-Ay mice a high-fat diet during a period of over 20 weeks. Animal body weight, fasting blood glucose (FBG), insulin tolerance, lipid metabolism, renal function, and renal pathology were periodically measured (once every 2 or 4 weeks) over a duration of 20 weeks. At the 12th week, an Affymetrix gene chip assay was performed on the renal tissues extracted from the T2DM animal models and control animals. Through screening for the differentially expressed genes, some key genes were selected for PCR validation.

**Results:**

High level of glucose could inhibit the growth of kidney cells. Besides, KK-Ay mice were found to have high FBG and abnormal insulin tolerance. Renal dysfunction and pathology were observed at the 4th week following the start of model creation, which increased in severity over the length of the experiment. The T2DM SD rats also showed high FBG, abnormal glucose tolerance and abnormal lipid metabolism, but the renal function and renal pathology changed only slightly within 20 weeks. Gene profiling in animal kidneys and subsequent analyses and validation revealed differentially expressed genes and enriched pathways in DN.

**Conclusion:**

KK-Ay mice with both high fasting glucose and insulin resistance were more likely to develop diabetic nephropathy than STZ-induced diabetic SD rats with low fasting glucose or only insulin resistance. The KK-Ay mice model showed earlier onset of the typical pathological characteristics associated with T2DM and obvious renal lesions suggestive of kidney damage.

## Introduction

Diabetes mellitus (DM) is a chronic disease caused by relative or absolute insufficiency of insulin secretion, a decrease in insulin sensitivity of target cells or structural defects in insulin itself, resulting in metabolic disorders (DeFronzo et al., 2015; Gheith et al., 2015). There are currently 400 million people with diabetes worldwide and the number of DM patients is increasing at a rate of 4 million per year (Wild et al., 2004; Forouhi and Wareham, 2014; Martínez-Castelao et al., 2015). Persistent hyperglycemia, insulin resistance and lipid metabolism disorders can lead to a series of complications such as diabetic retinopathy, diabetic nephropathy, diabetic foot and neuropathy (American Diabetes Association, 2009; Terry et al., 2012; Chawla et al., 2016).

Diabetic nephropathy (DN) is one of the most serious and common microvascular complications, and it is also one of the main causes of end-stage nephropathy and renal failure (Dronavalli et al., 2008). It is characterized by glomerular vascular injury, glomerulosclerosis, the formation of nodular lesions and deteriorating renal function that eventual leads to end-stage renal disease (Schena and Gesualdo, 2005; Kanwar et al., 2011). The incidence of diabetic nephropathy is about 33–40% among type 1 diabetics and 20–25% among type 2 diabetes (Khwaja et al., 2007; Olokoba et al., 2012). In recent years, clinical and experimental studies have shown that the pathogenesis of diabetic nephropathy is very complex. A growing number of studies have shown that glucose metabolism disorders, the formation of glycosylation end products, the activation of polyol pathway (Yamagishi et al., 2007), the increased activity of protein kinase C (Gnudi et al., 2003) and the changed renal hemodynamics collectively play an important role in the pathogenesis of diabetic nephropathy. Meanwhile, cytokines changed (Hellemons et al., 2012; Hao et al., 2013; Nakagawa et al., 2013), lipid metabolism disorders (Attman et al., 1998), genetic susceptibility (Schena and Gesualdo, 2005), oxidative stress (Forbes et al., 2008) and other factors are also involved.

Multiple DN animal models including those created by surgical excision of pancreas or chemical induction, spontaneous DN animals and transgenic DN animals have been used for the study of DN pathogenesis and the development of anti-DN drugs (King, 2012; Al-Awar et al., 2016). Two approaches to establish animal models of type 2 diabetes mellitus (T2DM) have been widely used: chemical induction with drugs such as streptozotocin (STZ) combined with high fat feeding in rats and spontaneous animal models with gene mutation like KK-Ay mice. STZ is a compound that has a preferential toxicity toward pancreatic β cells (Lenzen, 2008). The KK-Ay mouse was produced by transferring the yellow obese gene (Ay allele) into the KK/Ta mouse that spontaneously exhibited T2DM phenotypes associated with hyperglycemia, glucose intolerance, hyperinsulinemia, mild obesity and microalbuminuria (Suto et al., 1998; Tomino, 2012). The phenotypic characteristics of different types of T2DM models are different (King, 2012; Al-Awar et al., 2016), which indicates that the pathogenesis of DN, such as the onset, progress speed and degree of renal lesions in these models, might also be different. Therefore, the study of the characteristics and mechanisms of renal lesions using different T2DM animal models would likely contribute to the selection of suitable animal models for developing anti-DN drugs.

In this study, we established an animal model of T2DM SD rats using low dose STZ combined with high fat feeding and another animal model of spontaneous T2DM in KK-Ay mice with high fat feeding alone. The dynamic changes of glucose and lipid metabolic parameters, renal function-associated parameters, and pathological changes of kidneys along with the observation periods of 20 weeks in both T2DM SD rats and KK-Ay mice and their corresponding control animals were compared in a pairwise fashion. In addition, the molecular mechanisms underlying the differed pathogenesis of DN in these two models were preliminarily explored by gene profiling of renal tissues with microarrays.

## Materials And Methods

### Cell Culture

Human renal proximal tubular cell line (HKC) and the stable MES 13 murine mesangial cells transformed with non-capsid-forming SV 40 virus were obtained from Institute of basic medicine, Chinese academy of medical sciences. All the cell lines were cultured in DMEM culture medium with 10% FBS and in a humidified atmosphere of 5% CO_2_ at 37°. They were digested by 0.25% trypsin until they reached approximately 80% confluence. Cells that were after passages 2 were used in this study.

### Measurements of Cell Growth Inhibition

After the digestion by trypsinization, the cell concentration of every cell line was adjusted to 2 × 10^4^/mL and inoculated in three 96-well plates. Every hole contained 200 μL cell suspension solution or PBS. Each 96-well plates inoculated 50 holes which were divided into 10 groups, besides, PBS (200 μL in every hole) was add in the holes around them and the plates were cultured in a humidified incubator containing 5% CO_2_ at 37°C. After 24 h, the medium of different groups was replaced with new medium containing different sugar concentrations and all the plates continued to culture in incubator for different time (24, 48, 72 h). At every point in time, each hole added 20 μL (5 mg/mL) MTT and put them back in the incubator for another 4 h. After that, the supernatant was abandoned and each hole added 150 μL DMSO and mixed by microporous plate thermostatic shaker for 15 min. The 96-well plates were placed in full spectrum enzyme marker at 570 nm for measuring the inhibition ratio.

$$\begin{array}{c} \mathrm{Inhibition ratio}\;(\%)=(\mathrm{control group}-\mathrm{treatment group})\\ \;/(\mathrm{control group}-\mathrm{background group})\\ \;\times 100\% \end{array}$$

### Animals

This study was performed in strict accordance with the recommendations in the Guide for the Care and Use of Laboratory Animals of the National Institute of Health. All procedures involving experimental animals were approved by the University Committee on the Use and Care of Animals (UCUCA) at the Beijing Yizhuang Biomedical Park Animal Center (approval number 2017S007).

Specific-pathogen-free (SPF) grade healthy SD rats (male, 170—190 g), C57BL/6J mice (male, 8 weeks old) and KK-Ay mice (male, 8 weeks old) were purchased from Beijing Huafukang Biotechnology Co. Ltd. (Beijing, China). The animals were housed at the experimental animal center of Beijing Yizhuang Biomedical Park Animal Center with a controlled temperature (22 ± 2°C) and humidity (50% ± 5%) on a 12 h alternate light-dark cycle. Food and water were provided *ad libitum* throughout the experiments.

The number of experimental animals in this study was calculated based on the study design of published related papers. We used the minimum sample size at each time point on the premise of ensuring statistical differences, to be specific, we included at least 6 animals at each time point for biochemical markers, urine analysis, body weight, et al. (Tahara and Takasu, 2018), and 3 animals of each group for kidney sampling and subsequent pathology (qualitative investigation) and sequencing at different time-point (Conesa et al., 2016).

### Induction Methods of Type 2 Diabetic Models

Type 2 DM rats were induced by low dose STZ administration and high-fat-diet feeding (Gheibi et al., 2017). SD rats were randomly divided into “Control Rats” group (*n* = 15) and “T2DM Rats” group (*n* = 54).

**FIGURE 1:**
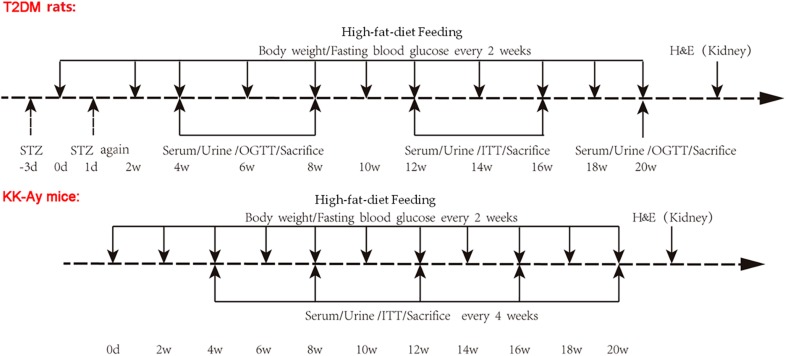
Schematic representation of experimental design. Type 2 DM rats were induced by low dose STZ administration and high-fat-diet feeding. SD rats were randomly divided into Control rats group and T2DM rats group. After fasting for 10 h, rats in the T2DM rats group were intraperitoneally injected with 1% STZ citrate buffer at a dose of 30 mg/kg body weight in the first time (−3d). At 72 h after STZ injection, these rats were fasted for 3 h and subjected to measurements of FBG. The rats with FBG < 11.1 mmol/L were re-injected with the same dose of 1% STZ on the next day (1d). FBG was measured after 72 h and continued to be observed for 20 weeks. Type 2 DM rats were fed with high-fat-diet starting day 0. Rats in the Control rats group were injected with the same amount of citrate buffer, and were fed with normal diet. The KK-Ay mice model of spontaneous type 2 DM was established by feeding with high-fat-diet which was started at day 0, while the C57BL/6J control mice were fed with normal diet. The body weight and FBG for rats and mice were recorded every 2 weeks. Serum and urine samples were collected, and OGTT and ITT were performed, at the indicated time points after the initial of modeling. Animals were sacrificed at the indicated time points, and renal tissues were used for H&E staining. STZ, streptozotocin; FBG, fasting blood glucose; OGCT, oral glucose challenge test; ITT, insulin tolerance test.

After fasting for 10 h, rats in T2DM Rats were intraperitoneally injected with 1% STZ (Lot number WXBC3087V, Sigma-Aldrich, St. Louis, MO, United States) citrate buffer (pH = 4.2–4.5) at a dose of 30 mg/kg body weight in the first time (−3d, Figure 1). At 72 h after STZ injection, these rats fasted for 3 h before measurements of fasting blood glucose (FBG) were taken. The rats with FBG < 11.1 mmol/L were re-injected with the same dose of 1% STZ on the next day (1d, Figure 1). FBG was again measured after 72 h and continued to be measured for 20 weeks. Type 2 DM rats were fed with a high-fat diet (10% lard + 20% sucrose + 2.5% cholesterol + 1% cholate + 66.5% conventional chow) during the whole experimental process (Gheibi et al., 2017). Rats in the Control rats group were injected with the same amount of citrate buffer, and were fed with a normal diet.

Male C57BL/6J mice (*n* = 18) and KK-Ay mice (*n* = 18, SPF grade, 8 weeks old) were maintained in the individually ventilated cages (IVC) at the experimental animal center for 20 weeks (Figure 1). The KK-Ay mice model of spontaneous type 2 DM was established by feeding with high-fat diet (protein 16.5 kcal%, carbohydrate 37.9 kcal%, fat 45.6 kcal%) (Gheibi et al., 2017), while the C57BL/6J control mice were fed a normal diet. All feed was purchased from Beijing Huafukang Biotechnology Co. Ltd. with a license No. SCXK (Beijing) 2014-0008.

### Biochemical Indicators Test

The body weight and FBG for rats and mice were recorded every 2 weeks. In the rat groups, the oral glucose tolerance test (OGTT) was performed at weeks 4, 8, and 20, while the insulin tolerance test (ITT) was performed at weeks 12 and 16. In the mouse groups, OGTT and ITT were performed every 4 weeks starting from week 4 (Figure 1).

The concentrations of urinary protein and urinary microalbumin were determined every 4 weeks starting from week 4, and 24 h urinary albumin excretion rate (UAE) was calculated (Figure 1). For urine samples collection, the beddings in cages were removed and homemade urine filters were placed on the bottom of the cages. After 24 h, urine at the bottom of the cage was collected with a disposable pipette and the amount of urine was recorded. After statically settled down overnight at 4°C, the supernatant was taken for detecting urine related indicators.

Every 4 weeks, three animals from the control groups and another three from the diabetic groups were randomly selected to measure the biomedical indicators using various kits. The following kits were from Nanjing Jiancheng Bioengineering Institute, Nanjing, China: Creatinine determination kits (batch number 20170209), albumin kit (batch number 20170222), blood urea nitrogen (BUN) kits(batch number 20170522), triglyceride (TG) kits (batch number 20170221), total cholesterol (T-CHO) kits (batch number 20170222), low-density lipoprotein-cholesterol (LDL-C) kits (batch number 20170519). Urine protein kit (batch number 20170821) and urinary microalbumin kit (batch number 20170227). The quantitation of these parameters was performed following the manufacturer’s protocols. Blood glucose was monitored using blood glucose meter (Roche, United States). Glucose (batch number 20170110) was obtained from Beijing Boaoxing Biotechnology Co., Ltd. (Beijing, China), and recombinant human insulin injection (Humulin, R) (batch number C553562D) was obtained from Lilly France.

### Hematoxylin-Eosin Staining of Kidney

The unilateral kidney tissues of animals in the diabetic model group and the corresponding control group were extracted once every 4 weeks. The kidneys were fixed in 4% paraformaldehyde for 3 days and the following routine procedures were performed on the coronal tissue blocks, including dehydration, fixation, and paraffin embedding, and sectioning (3 μm). The sections were stained with hematoxylin and eosin (H&E) after dewaxing and rehydration. Pathological changes of kidney were observed under a light microscope. Three view fields were randomly selected for each slide, and were scored independently by two pathologists.

### Gene Expression Profiles With Affymetrix 3′IVT Gene Chips

GeneChip^TM^ Rat Genome 230 2.0 Array chip and GeneChip^TM^ Mouse Genome 430 2.0 Array chip (Affymetrix, Inc., Santa Clara, CA, United States) were used to profile gene expressions of rat and mouse kidney tissues, respectively. Total RNA samples were extracted with the TRIzol reagent (Invitrogen, Carlsbad, CA, United States), and then quantified using a spectrophotometer. The integrity of RNA samples was tested by agarose gel electrophoresis. The samples with total RNA amount >1 μg, A260/A280 ≥ 1.80 and a brightness ratio of 28 s band: 18 s band greater than or close to 1:1 were judged to be samples qualified for microarray. The chip hybridizations were carried out following the manufacturer’s specifications. The AGCC software was used to save the chip fluorescence scanning image into a.DAT file for analysis (Schroeder et al., 2006; Villegas-Ruiz et al., 2016).

### Data Processing Methods of IVT Gene Expression Profiles

The results of gene chip were analyzed and plotted by R-Project software, and the data quality of gene chip was evaluated using chip signal intensity distribution statistics and relative logarithmic signal intensity statistics. The probes with the lowest 20% of signal intensity tested in all samples were filtered out as background noise. Genes with significant differential expression were screened according to the criteria of differential fold exchange ≥ 2.0 and *q* ≤ 0.05. The above significant differentially expressed genes were classified by biological process, molecular function and cellular component aspect in the enrichment analysis by means of gene ontology (GO) annotation enrichment analysis with the software, CapitalBio^®^ Molecule Annotation System V3.0. The significant enrichment level of a gene was evaluated by Fisher accurate test (Masuda et al., 2017).

### Quantitative Real-Time PCR

Total RNA was extracted from the frozen kidneys with RNApure kit (Bioteke, Beijing, China) according to the instructions of the manufacturer. Strand cDNA was synthesized by Plus All-in-one 1st Strand cDNA Synthesis SuperMix kit (NovoScript, Beijing, China) according to the instructions of the manufacturer. Twelve genes were selected to analyze their mRNA expression differences. The primers of these genes were listed in Table 1. The expression levels of genes were measured by 7500 FAST Real-Time PCR System (Thermo Fisher Scientific, United States). Gene expression levels were performed by 2 × Plus SYBR real-time PCR mixture according to the instructions of the manufacturer and quantified relatively to the expression of the GAPDH by using an optimized comparative Ct (△△Ct) value method which was calculated as 2^∧^ (△△Ct) to compare the relative expression.

**TABLE 1:** The primers of Twelve genes.

Gene	Primer sequences	Gene No.
Rat TGF-β F	5′-CAAGCAGAGTACACACAGCA-3′	59086
Rat TGF-β R	5′-GATGCTGGGCCCTCTCTCCAGC-3′	
Rat CTGF F	5-AAGACCTGTGGGATGGGC-3	64032
Rat CTGF R	5-TGGTGCAGCCAGAAAGCTC-3	
Rat PPARγ F	5-CAT AAA GTC CTT CCC GCT GA-3	25747
Rat PPARγ R	5-GAA ACTGGC ACC CTT GAA AA-3	
Rat P38 F	5′-CTGCGAGGGCTGAAGTAT-3′	81649
Rat P38 R	5′-TCCTCTTATCCGAGTCCAA-3′	
Rat Erk F	CTACACGCAGCTGCAGTACATC	24338
Rat Erk R	GTGCGCTGACAGTAGGTTTGA	
Rat Akt F	5′-TCAGGATGTGGATCAGCGAGA-3′	24185
Rat Akt R	5′- CTGCAGGCAGCGGATGATAA-3′	
Rat VEGF F	5′-CCACTTCTGTCTTGCCACACA-3′	83785
Rat VEGF R	5′-CCAACCAATTAAGACCTTCTG-3′	
Rat RAGE F	5- CAGGGTCACAGAAACCGG-3	81722
Rat RAGE R	5′-ATTCAGCTCTGCACGTTCCT-3′	
Rat β-actin F	3-GTAAAGACCTCTATGCCAACA-5	81822
Rat β-actin R	3-GGACTCATCGTACTCCTGCT-5	
Rat PI3K F	5′-CCCATGGGACAACATTCCAA-3′	25513
Rat PI3K R	R 5′-CATGGCGACAAGCTCGGTA-3′	
Rat Fst F	5′-GTGTATCAAAGCAAAGTCTTG-3′	24373
Rat Fst R	5′-GCTCATCGCAGAGAGCA-3′	
RatOPN_1F	5-AGGTCATCCCAGTTGCC-3	81644
RatOPN_1R	5-GGCCCTCTGCTTATACTCC-3	

The whole process of biochemical indicators test, Hematoxylin-eosin staining and gene expression profiles of kidney at different time point of animal model of T2DM is shown in Supplementary Table 1.

### Statistical Analysis

Data were analyzed by SPSS 16.0 software (IBM; Armonk, NY, United States), and were expressed as mean ± standard deviation (SD). The one-way analysis of variance (ANOVA) was used to compare the differences among groups. A *P* < 0.05 was considered to be statistically significant.

## Results

### The Inhibition of Different Concentration of Glucose on Cell Growth

**FIGURE 2:**
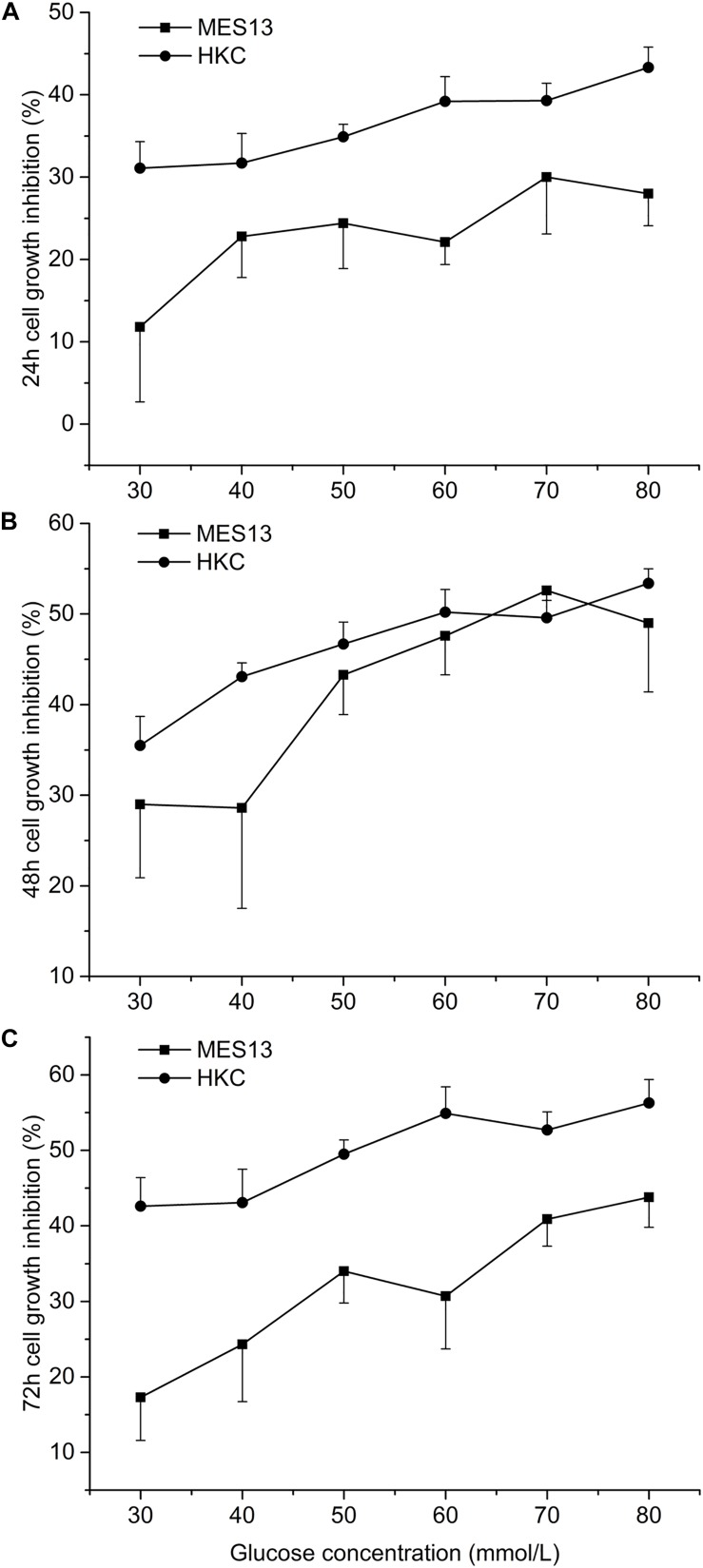
Effects of different concentration of glucose on cell growth. After cell inoculation, cells were cultured for 24 h at incubator. Then each hole was added different concentration of glucose into the cell supernatant for 24 h **(A)**, 48 h **(B)**, or 72 h **(C)**. At the selected time point, the 96-well plate was taken out form the incubator for measuring the inhibition rate.

The inhibition of cell growth gradually increased with the increase of glucose concentration, but the maximum inhibition rate was less than 60%. On MES13, the inhibitory effect of different concentrations of glucose on cells achieved maximum at 48 h and then decreased (Figures 2A–C). However, this inhibition rate reached maximum at 72 h on HKC (Figure 2C). This experiment also showed that HKC was more sensitive to glucose concentration and duration than MES 13.

### The Dynamic Changes of Glucose and Lipid Metabolic Parameters in T2DM Rats and KK-Ay Mice

**FIGURE 3:**
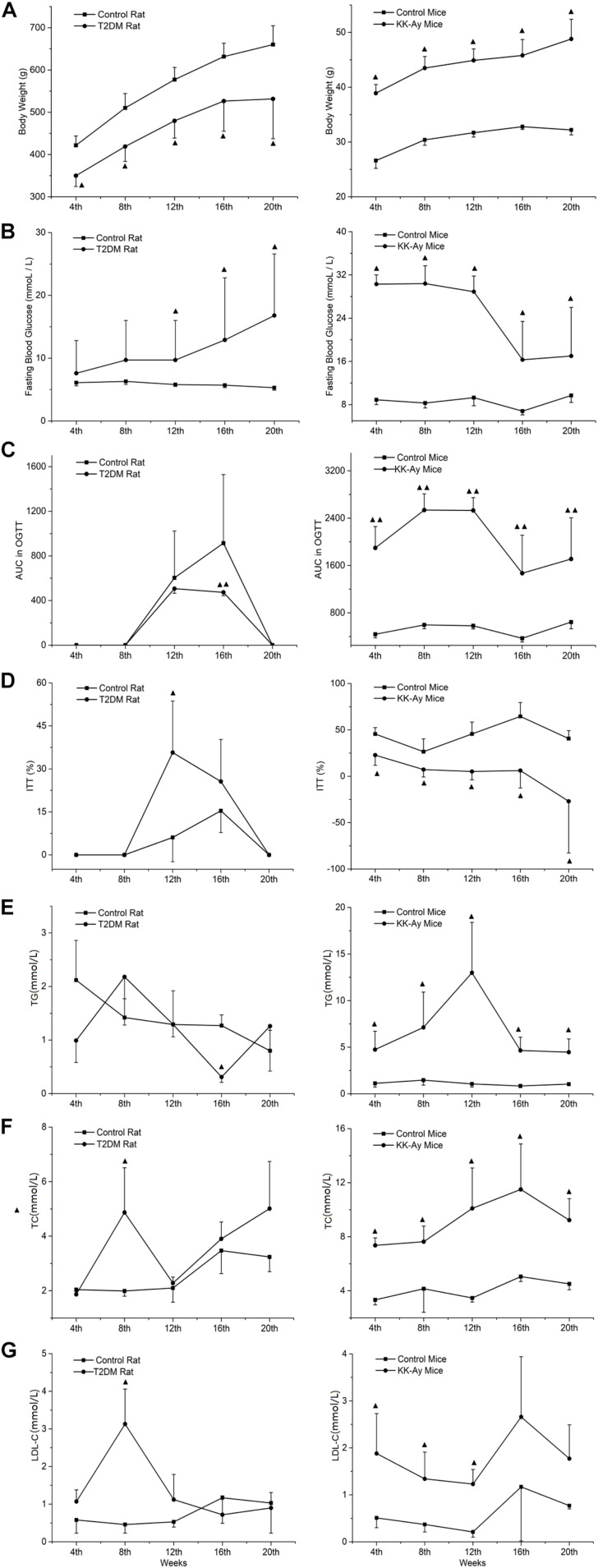
Comparisons on the dynamic changes of glucose and lipid metabolic parameters in T2DM rats and KK-Ay mice. **(A–G)** The dynamic changes of body weight **(A)**, FBG **(B)**, AUC in OGCT **(C)**, percentage of ITT **(D)**, TG **(E)**, TC **(F)**, and LDL-C **(G)** in T2DM rats (left) and KK-Ay mice (right) along with the indicated time points after the initial of modeling are shown. ▲*P* < 0.05, ▲▲*P* < 0.01, compared with the corresponding control group at the same time point. FBG, fasting blood glucose; OGCT, oral glucose challenge test; ITT, insulin tolerance test; AUC, area under the curve; TG, triglyceride; TC, total cholesterol; LDL-C, low-density lipoprotein-cholesterol.

In rats with T2DM induced by low-dose STZ combined with high fat feeding, the glucose and lipid metabolism associated biochemical indexes were measured at different time points within 0–20 weeks after modeling. The body weight of the T2DM rats was significantly lower than that of the control rats at the all time points from week 4 to week 20 (*P* < 0.05; Figure 3A). A transient increase in FBG was observed after 3 days of the second injection of STZ (14.1 ± 6.9 mmol/L) in T2DM rats, and it remained at a low level during the next 4–8 weeks time points (FBG < 11.1 mmol/L). At the 12–20 weeks time points, a significant increase of FBG was identified (FBG > 11.1 mmol/L) (P < 0.05; Figure 3B). The OGTT results showed that at the 12th week, the percentage of blood glucose and the area under the curve (AUC) increased significantly (*P* < 0.05, data not shown) in 30 min in the T2DM rats after orally taking glucose solution when compared with the control group, and it also kept at a higher level at the 16th week (*P* < 0.05; Figure 3C). The ITT results showed that at the 12th week, the reduction percentage of blood glucose in 40 min after subcutaneous injection of insulin in the T2DM rats was significantly higher than that of the control rats. There was a slight ITT abnormality but no significant difference between the model and the normal rats at the 16th week (Figure 3D). Compared with the control rats, the T2DM rats demonstrated transient decrease of TG (Figure 3E) at week 16, and transient increase of TC (Figure 3F) and LDL-C (Figure 3G) at week 8 (all *P* < 0.05).

Compared to the control C57BL/6J mice, the body weight of KK-Ay mice increased significantly from 4 to 20 weeks after starting high-fat-diet feeding (Figure 3A). At week 4 there was a significant increase in FBG (*P* < 0.05) in the KK-Ay mice, and FBG values remained at 30 mmol/L during the first 12 weeks. Starting from week 16, the FBG values dropped to about 17 mmol/L (Figure 3B). Four weeks after modeling, OGTT results in the KK-Ay mice always showed a higher AUC value than the control mice (Figure 3C). The results of ITT showed that the reduction percentage of blood glucose in the model group was significantly less than the control group at all the time points between weeks 4 and 20 (*P* < 0.05; Figure 3D), which indicates an obvious insulin resistance phenomenon. The lipid metabolism indexes (TG, TC, and LDL-C) in the KK-Ay mice were significantly higher than those in the C57BL/6J mice starting from the 4th week (*P* < 0.05), and the difference between the two groups was most significant at the 12th week (Figures 3E–G). There was a recovery tendency of lipid metabolism over time, but the KK-Ay mice still have significantly higher lipid metabolism indexes (TG and TC) after the 16th week, compared with the C57BL/6J mice.

### The Dynamic Changes of Renal Function-Associated Parameters in T2DM Rats and KK-Ay Mice

**FIGURE 4:**
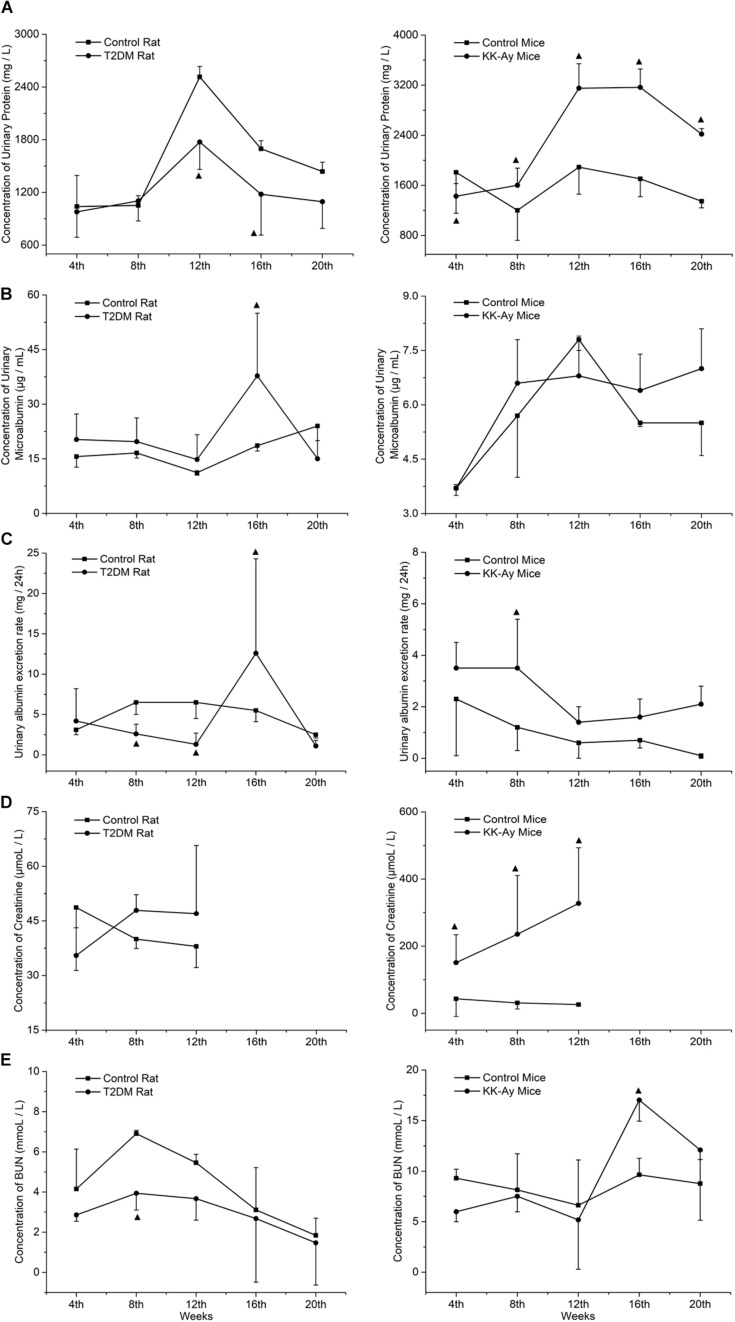
Comparisons on the dynamic changes of renal function-associated parameters in T2DM rats and KK-Ay mice. **(A–E)** The dynamic changes in concentrations of urinary protein **(A)**, concentrations of urine microalbumin **(B)**, urinary albumin exclusion rate (UAE) **(C)**, concentrations of serum creatinine **(D)**, and concentrations of urea nitrogen **(E)** in T2DM rats (left) and KK-Ay mice (right) along with the indicated time points after the initial of modeling are shown ▲*P* < 0.05, compared with the corresponding control group at the same time point.

Renal function was evaluated by dynamic detection of urine biochemical indexes in the T2DM animal models and the normal control animals. Compared to the Control rats at the same time points, the urinary protein concentrations of T2DM rats decreased significantly at the 12th and 16th week (*P* < 0.05; Figure 4A). The concentration of urine microalbumin in T2DM rats increased significantly at the 16th week (*P* < 0.05; Figure 4B). Urinary albumin exclusion rate (UAE) also decreased significantly at the 8th and 12th week in T2DM rats, but it increased accidentally at the 16th week (Figure 4C). The concentrations of serum creatinine (Figure 4D) and urea nitrogen (Figure 4E) in T2DM rats were not significantly different from those in the control group at each time point, except that T2DM rats had significantly lower urea nitrogen levels at the 8th week (Figure 4E).

Compared to the control C57BL/6J mice at the same time points, the urine protein concentration (Figure 4A) and serum creatinine concentration (Figure 4D) of KK-Ay mice increased significantly (*P* < 0.05) and gradually increased in first 12th weeks. Urea nitrogen concentration in KK-Ay mice had a transient increase at the 16th week (*P* < 0.05; Figure 4E) and the urinary albumin exclusion rate (UAE) increased significantly at the 8th week (*P* > 0.05; Figure 4C), but there was no significant difference in terms of urinary microalbumin concentration (Figure 4B) between the two groups.

### Comparisons on the Pathological Changes of Kidney in T2DM Rats and KK-Ay Mice

**FIGURE 5:**
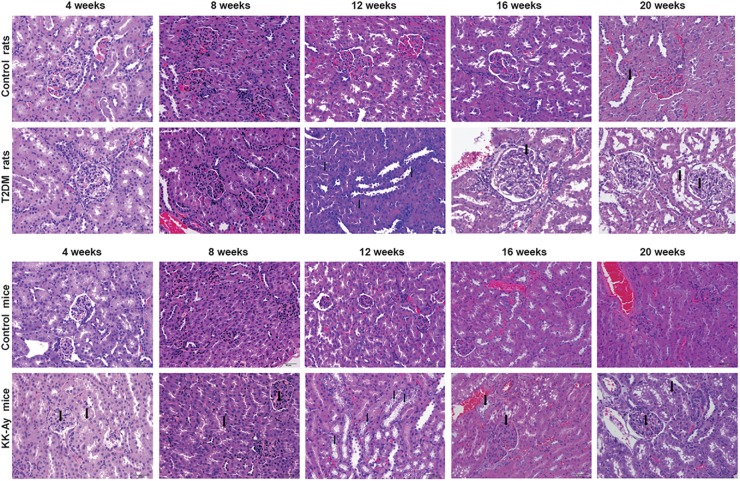
Comparisons on the pathological changes of kidney in T2DM rats and KK-Ay mice. Representative H&E images show the pathological changes of kidneys from control/T2DM rats and control/KK-Ay mice at the indicated time points after the initial of modeling. Magnification, 40×.

Renal pathology was examined at different time points in different T2DM models and the corresponding control groups by H&E staining. At 4 and 8 weeks after the start of model creation, there was no obvious pathological change in renal tissue of rats with T2DM, compared with the Control rats (Figure 5). At the 12th week, there was slight hyperplasia of glomerular mesangial cells in the kidney of one animal in the model group. At the 16th week, the renal histopathological changes associated with diabetic nephropathy were observed in T2DM rats, such as glomerular mesangial cell proliferation, glomerular mesangial matrix increase and the clear and empty cytoplasm of renal tubular epithelial cells (Figure 5). In addition to the above pathological changes, glomerular hypertrophy and thickening of glomerular capillary basement membrane were observed in the kidney of T2DM rats at the 20th week (Figure 5). The changes described above are typical pathological changes in the kidneys of diabetic animals. In addition, in each stage of modeling, a few kidneys, whole or in part, including those in the Control rats and T2DM rats, could be identified to display the renal tubule lumen protein flocculation, renal tubule dilatation, chronic progressive nephropathy (CPN, manifested as tubule basophilic degeneration), and pyelonephrosis, and these lesions are common spontaneous lesions in SD rats. There was no significant difference in the incidence and severity of these lesions between the control group and T2DM group (Table 2).

**TABLE 2:** Pathological changes of kidney in different diabetes mellitus models.

Group	Time (week)	Increase of glomerular mesangial matrix	Vacuolar degeneration of cortical renal tubular epithelial cells	Tubular epithelial cells cytoplasmic shell	Tubular protein	InterstItial inflammatory cell infiltration	Basophilic degeneration of renal tubule	Distal convoluted tubule dilatation	Pyelectasia
Control rat	4th	–	–	/	–	/	±	/	–
	8th	–	–	–	–	–	–	–	–
	12th	–	–	–	–	–	–	–	–
	16th	–	–	–	–	–	+	±	–
	20th	–	–	–	–	–	+	±	–
T2DM rat	4th	–	–	/	–	/	–	/	Y
	8th	–	–	–	–	–	–	–	–
	12th	+	2+	2+	–	–	+	+∼2+	–
	16th	+	2+	2+	–	–	+	+	–
	20th	+	+	+	–	–	+	+	–
Control mice	4th	–	–	/	±	/	–	/	–
	8th	–	–	–	–	–	–	–	–
	12th	–	+	–	–	–	–	–	–
	16th	–	+	–	–	–	–	–	–
	20th	–	+	–	–	–	–	–	–
KK-Ay mice	4th	2+∼3+	2+	/	+	/	+	/	–
	8th	2+	+	–	+	+	+	–	Y
	12th	2+∼3+	+∼2+	+	+∼2+	+	+∼2+	+	Y
	16th	2+∼3+	+∼3+	+	–	–	–	–	3 +
	20th	3+	+∼3+	2+	–	–	–	–	3 + ∼4 +

*N = 3∼5. ±, suspicious or extremely minor lesion; +, minor lesion; 2+, mild lesion; 3+, moderate lesion; 4+, severe lesion; Y, pathological changes regardless of grade;/, not determined. Data in the list was obtained from individuals with the most serious grade of pathological changes.*

Compared to the control C57BL/6J mice at the same time points, the increase in mesangial matrix, the tubular epithelial cells vacuolar degeneration and CPN were observed in the kidney of KK-Ay mice during the full period (Figure 5). After 8 weeks of high fat feeding, some of the KK-Ay mice had the kidneys with renal pelvis dilatation. In the KK-Ay mice after 16 weeks of modeling, besides above lesions, glomerular hypertrophy, glomerular capillary basement membrane thickening, glomerular mesangial cell proliferation and glomerular segmental sclerosis were observed in their kidneys (Figure 5). At the 20th week, renal tubular epithelial cells in KK-Ay group were empty and blank, while tubule dilatation and protein flocculation in renal tubules were observed. In the above lesions, glomerular hypertrophy, thickening of glomerular capillary basement membrane, proliferation of glomerular mesangial cells, increase of glomerular mesangial matrix and glomerular segmental sclerosis were the characteristic pathological changes of diabetic kidney. Vacuolar degeneration of cortical renal tubular epithelial cells, CPN (characterized by basophilic degeneration of renal tubule, renal tubular hyaline and interstitial inflammatory cell infiltration), renal tubule dilatation, tubule protein flocculation and pelvis dilatation may be spontaneous lesions. The incidence of these lesions in the kidney of KK-Ay mice tended to be increased, compared with that in the control C57BL/6J mice (Table 2).

### Comparisons on the Changes of Gene Expression Profiles in T2DM Rats and KK-Ay Mice

**FIGURE 6:**
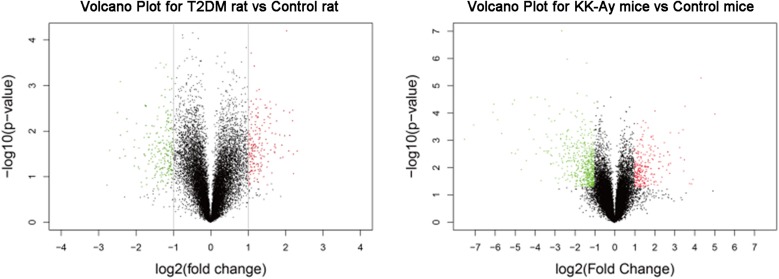
Analyses of differentially expressed genes in kidneys from the T2DM animal models. Volcano plots of differentially expressed genes in the renal tissues from T2DM rats **(left)** and KK-Ay mice **(right)** are shown. The fold changes are represented in log2 scale as depicted on the x-axis, whereas the −log10 *P*-value is depicted on the y-axis. Genes with greater statistical significance are highlighted in the plots. The red/green dots represent genes that show differential expression with fold changes > 2 (red) or < 0.5 (green) and *P*-values < 0.05 between the T2DM renal tissues and normal control renal tissues.

In order to reveal the molecular mechanisms underlying the pathological changes of kidneys in T2DM rats and KK-Ay mice, we performed gene expression profiling in kidney samples by Affymetrix 3′ IVT gene microarrays. As shown in the volcano plots in Figure 6, compared with the Control rats group, the T2DM rats group had 449 differentially expressed genes (—fold change— ≥ 2, *q* ≤ 0.05) in the renal tissues, among which 213 genes were up-regulated and 236 genes were down-regulated. Compared with the control C57BL/6J mice, KK-Ay mice had 1064 differentially expressed genes in the renal tissues, among which there were 334 up-regulated genes and 730 down-regulated genes. Of these, 73 differentially expressed genes were identified in both T2DM SD rats and spontaneous KK-Ay mice. The five most up-regulated genes and five most down-regulated genes in the renal tissues of T2DM SD rats and KK-Ay mice are listed in Table 3.

**TABLE 3:** Summary of differentially expressed genes in kidney tissue of diabetic nephropathy animals (fold change ≥ 2 or ≤ 0.5, *q*-value ≤ 0.05).

Comparison	Gene symbol	*q*-value (%)	Fold change
T2DM rat vs. Control rat	Fst	0.88	4.98
	Cftr	1.46	4.64
	Gja1	0	3.95
	Sod3	1.40	2.62
	Pcdh9	0.88	3.82
	Smlr1	0	0.17
	Naglt1	0.62	0.15
	Angptl4	0	0.20
	Igfbp1	1.04	0.29
	F2	0.19	0.21
KK-Ay mice vs. Control mice	Lox	0	32.54
	Ighg	0.37	30.63
	Mela	0	20.10
	Lcn2	0.22	15.24
	Adgra1	0.09	11.10
	Spink1	0	0.01
	Ttr	0	0.01
	Stk4	0	0.02
	Serpina1f	0	0.02
	Methig1	0	0.05

*Fold Change ≥2 means up -regulation; Fold Change ≤0.5 means down-regulation.*

Further analyses on the differentially expressed genes between the model groups and the control groups suggested that the biological pathways and molecular functions of these genes were different (Table 4). The differentially expressed genes in the T2DM SD rats were primarily related to the pathways of glucose and lipid metabolism disorder, amino acid metabolism and angiogenesis. The dysregulation of glucose and lipid metabolism is consistently a characteristic of T2DM. Abnormal amino acid metabolism and angiogenesis are also the main characteristics of diabetic nephropathy. The differentially expressed genes in KK-Ay mice are mainly related to the amino acid metabolism pathway disorder, insulin secretion abnormality, glucose and lipid metabolism disorder and impaired coagulation function observed in the mice. The relative insufficiency of insulin secretion and insulin resistance are also typical characteristics of T2DM. The change in coagulation function may affect the renal hemodynamics and lead to renal lesion.

**TABLE 4:** Enriched pathways from microarray detection of renal tissues in diabetic nephropathy.

DM	Enriched KEGG pathway	Input gene number
T2DM rat	Pentose and glucuronate interconversions	14
	Starch and sucrose metabolism	15
	Retinol metabolism	15
	Fatty acid degradation	7
	Beta-Alanine metabolism	6
	Tryptophan metabolism	6
	Complement and coagulation cascades	7
	Glycolysis/Gluconeogenesis	5
	Biosynthesis of amino acids	5
	PPAR signaling pathway	5
KK-Ay mice	PPAR signaling pathway	15
	Peroxisome	15
	Complement and coagulation cascades	14
	Tryptophan metabolism	9
	Protein digestion and absorption	13
	Retinol metabolism	11
	Fatty acid metabolism	8
	Fatty acid degradation	7
	Phenylalanine metabolism	4
	Arginine and proline metabolism	7

### Expression of Possible Key Gene Relating of the Mechanism of Diabetes Mellitus

**FIGURE 7:**
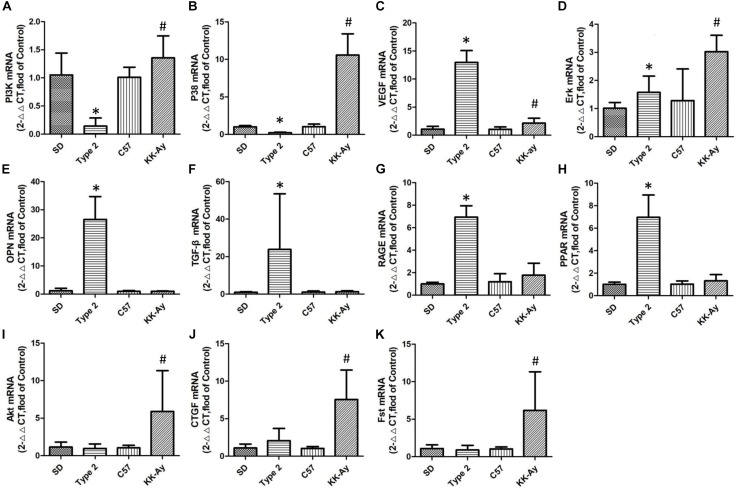
Relative mRNA levels of selected genes in T2DM rats and KK-Ay mice. Quantitative real-time PCR of the 5 samples were conducted 3 times and normalized to GAPDH gene expression, measured with 2^∧^ (−ΔΔCt) value. **(A)** PI3K mRNA, **(B)** P38 mRNA, **(C)** VEGF mRNA, **(D)** Erk mRNA, **(E)** OPN mRNA, **(F)** TGF-β mRNA, **(G)** RAGE mRNA, **(H)** PPAR mRNA, **(I)** Akt mRNA, **(J)** CTGF mRNA, and **(K)** Fst mRNA. Data are shown as the mean ± SE of 3 independent replicates. Significant differences (^∗^*P* < 0.05, or ^#^*P* < 0.05) between model group and corresponding control group.

In those selected genes, there were only two genes (Erk and VEGF) having the same tendency in the gene expression. These two genes increased to different degrees in their respective models. That means Erk and VEGF were the shared gene in different models of this study. Some gene, such as OPN, TGF-β, RAGE, PPAR, showed highly expression in T2DM SD rats other than KK-Ay mice. Similarly, Akt, CTGF, and Fst were proved to have significantly increased expression in mice. PI3K and P38 were more interesting than other genes, and revealed opposite direction in two models. The results were shown in Figure 7.

The expression of choiced genes not exhibited the same variation between two selected models. PI3K and p38 owned the contrary increase direction (*P* ¡ 0.05, *P*¡0.05), but VEGF and Erk had the same variation (*P* ¡ 0.05, *P* ¡ 0.05; Figure 7A). Although the other choiced genes also had the high expression, they only changed obviously in one of the two models (Figures 7B,C).

### The Predicted Interaction of Specific Proteins of Diabetes Mellitus Models

**FIGURE 8:**
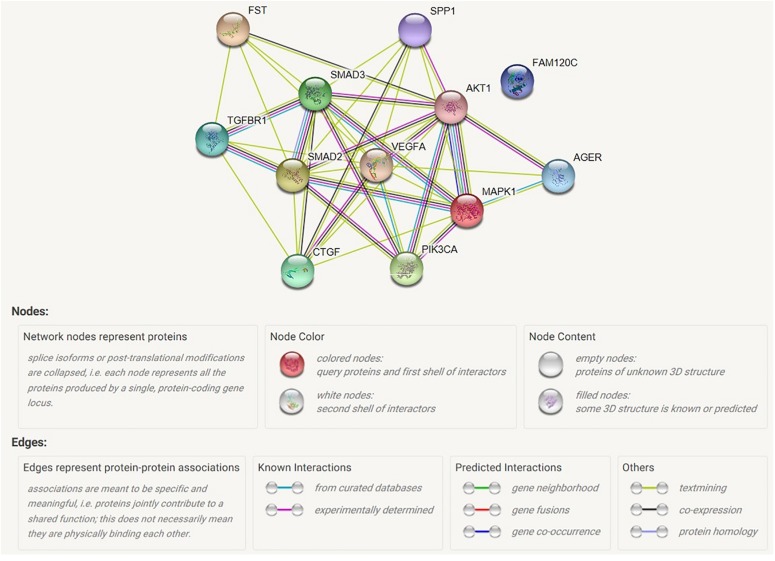
Map of possible interaction between selected genes. AGER, advanced glycosylation end product-specific receptor; AKT1, serine/threonine kinase 1; CTGF, connective tissue growth factor; FAM120C, family with sequence similarity 120C; FST, follistatin; MAPK1, mitogen-activated protein kinase 1; PIK3CA, phosphatidylinositol; SMAD2, mothers against decapentaplegic homolog 2; SMAD3, mothers against decapentaplegic homolog 3; SPP1, secreted phosphoprotein 1; TGFBR1, TGF-beta receptor type-1; VEGFA, vascular endothelial growth factor A.

The Raf/MAPK/ERK pathway and Ras/PI3K/AKT pathways are both involved in cell cycle regulation. When Ras active Raf and PI3K, the further kinase (ERK and AKT) could be active respectively. Receptor-regulated SMAD that is an intracellular signal transducer and transcriptional modulator active by TGF-βand activin type 1 receptor kinases. Bind the TRE element in the promoter region of many genes that are regulated by TGF-β and, on formation of the SMAD2/SMAD4 or SMAD3/SMAD4 complex, activate transcription. Also can form a SMAD3/SMAD4/JUN/FOS complex at the AP-1/SMAD site to regulate TGF-β-mediated transcription. Follistatin could bind directly to activin and functions as an activin antagonist. TGF-β-activated RhoA/ROCK signaling could induce Mesenchymal stem cells (MSCs) differentiate into myofibroblasts that promotes formation of an extracellular matrix (ECM) complex consisting of connective tissue growth factor (CTGF), and vascular endothelial growth factor (VEGF). Interestingly, CTGF and OPN have the closely negative correlation in the laryngeal squamous cell carcinoma although OPN might involve in cell-cell tight junction. AGER mediates interactions of advanced glycosylation end products (AGE). These are non-enzymatically glycosylated proteins which accumulate in vascular tissue in aging and at an accelerated rate in diabetes. The results were shown in Figure 8.

## Discussion

Diabetic nephropathy is one of the most common chronic microvascular complications in diabetic patients and it is also leading cause of end-stage renal disease (Dronavalli et al., 2008). Experiments using DN animal models are significantly facilitating the development of anti-DN drugs. However, no model currently exhibits all the features of human DN (King, 2012; Al-Awar et al., 2016). Therefore, biochemical, pathological, and transcriptomic data in selecting the appropriate model to study the molecules and pathways of interest should be considered (Betz and Conway, 2016). Besides, unstable onset time and uneven characteristics may significantly limit the stability of the models. Herein, we adopted the methods of inducing T2DM in rats by low-dose STZ combined with high fat diet and in KK-Ay mice with spontaneous type 2 diabetes. While examining the characteristics of DM, which included the dynamic changes of glucose and lipid metabolic parameters and renal function-associated parameters, we also studied the pathological changes in the kidneys during the whole process of renal lesion in these models. Furthermore, we explored the preliminary molecular mechanisms at the transcriptional level through gene profiling in renal tissues with microarrays. Our work provides an important reference for the evaluation of diabetic nephropathy models and contributes to our current understanding of the mechanisms of diabetic nephropathy of these T2DM animal models.

In our study, low-dose STZ combined with high fat feeding induced T2DM in SD rats, afterward which showed low FBG level and abnormal glucose tolerance. But there was no obvious abnormality in lipid metabolism and renal function. Renal lesions appeared in later time points and the main changes were glomerular mesangial matrix increasing, glomerular mesangial cell proliferation, glomerular hypertrophy and absence of tubular epithelial cells in cortex. The characteristics of renal lesions were closely related to insulin resistance. KK-Ay mice with spontaneous T2DM had the characteristics of high FBG, insulin resistance and abnormal lipid metabolism. For the renal function associated parameters, urinary protein excretion and serum creatinine were shown to have increased significantly. Renal lesions were displayed early onset and the main changes included increased glomerular mesangial matrix, glomerular mesangial cell proliferation, glomerular hypertrophy, glomerular capillary basement membrane thickening, glomerular segmental sclerosis, cortical renal tubular epithelial cells vacuolar degeneration, and CPN. The characteristics of renal lesions were closely related to high FBG, insulin resistance and abnormal lipid metabolism.

Different types of diabetes have different characteristics and pathogenesis that lead to different onset, progress speed and degree of renal lesions. Therefore, appropriate models should be selected to study the intervention of DN progression and to screen drugs for treating DN. Chemical induction of diabetes is widely used in DN animal models with the advantages of light injury, low mortality, high rate and low cost of model formation (Motshakeri et al., 2014). The animal model of spontaneous diabetic nephropathy reduces human factors to a certain extent, and the characteristics of renal lesions are similar to human DN. But the use of such model is limited due to its limited sources, high breeding conditions, low incidence of disease, long period of disease progression and high price (King, 2012; Al-Awar et al., 2016). Despite the essential differences in etiology of DM in the two animal models, our systemic in-depth analyses and comprehensive comparisons on the characteristics of DM and DN at different time points revealed some common factors of DN. For example, hyperglycemia was the key initial factor of DN, while renal hemodynamic changes and insulin resistance also played a key role in the formation of DN. In addition, hyperlipidemia also played a synergistic role in the occurrence and progression of DN.

### Hyperglycemia Is the Initiating Factor of Diabetic Nephropathy

High FBG and elevated postprandial blood glucose are the main characteristics of diabetes mellitus. When the blood glucose concentration in the body is too high, the pressure on glucose metabolism in the kidney increases, leading to an increase in glomerular filtration rate and renal tubular reabsorption, this changes the biochemical composition of glomeruli, increases renal vascular permeability, causes the leakage of plasma protein, and leads to renal tubule sclerosis and proteinuria (Fukami et al., 2004). The results showed that the FBG in the model of KK-Ay mice continuously kept at a high level (25–30 mmol/L) until the 12th week after modeling initiation, and the metabolism of glucose in kidney was increased due to the severe hyperglycemia in the body, which leads to the increase of glomerular filtration rate, tubular reabsorption and vascular permeability. Therefore, urinary protein and urinary albumin were found to have significantly increased 4 weeks after the onset of hyperglycemia, which was accompanied with typical pathological changes of DN such as mild cortical tubular epithelial cell vacuolar degeneration and tubular basophilic degeneration.

Chronic hyperglycemia increases the activity of enzymes associated with glucose metabolism, resulting in the formation complexes consisting of enzymes and proteins; these deposit in the glomeruli, resulting in serious damage (Miyata et al., 1998). In addition, the increase of glucose concentration can also promote the non-enzymatic glycosylation of non-glycosylated substances to produce advanced glycosylation end-products (AGEs) (Goh and Cooper, 2008). The kidney is the main organ that clears AGEs. AGEs can mediate the production of Ang2 by binding to the specific receptor on mesangial cells. It also leads to the synthesis and secretion of a large number of cytokines, such as interleukin (IL), insulin-like growth factor (IGF) and transforming growth factor (TGF-β), which leads to cell hypertrophy, fibronectin synthesis and glomerular sclerosis. Our results from gene profiling consistently showed that there were observable abnormalities in amino acid synthesis and metabolic pathways in the two DM animal models, which indicated that the kidney had functional abnormalities. In KK-Ay mice, abnormal metabolism of starch and glycogen occurred, indicated by the excessive expression of SMAD and PPARγ in the PPAR signaling pathway (Dutchak et al., 2012). Moreover, the analysis of differential genes of these diabetic models has shown significant increases in the expression of IGF, Ang, and ICAM in renal tissues.

In conclusion, the FBG of KK-Ay mice remained high for a long time, and compared to the T2DM SD rats, the renal lesions for these mice appeared earlier and were more severe. A high degree of proteinuria, vacuolar degeneration of renal tubular epithelial cells, basophilic degeneration and other pathological changes appeared at the 4th week after the initiation of the model, and with the extension of time, the rate and degree of pathological changes became increasingly faster and more severe. At the 8th week, the UAE index increased significantly, and according to the stage of Mogensen diabetic nephropathy, it can be classified as the stage 3. At every time point after modeling initiation, FBG level in the T2DM SD rats was lower than that in the KK-Ay mice. Therefore, onset time and degree of renal lesions in KK-Ay mice were more obvious than those in T2DM SD rats, which suggested that hyperglycemia was the initiator of diabetic nephropathy.

### Insulin Resistance Is an Important Factor Causing Diabetic Nephropathy

The main cause of T2DM is the relative insufficiency of insulin secretion or the decrease of insulin sensitivity. The direct consequence of the deficiency of insulin secretion is the decrease of glucose uptake and utilization, which leads to increased postprandial blood sugar (DeFronzo et al., 2015). Insulin resistance causes bodily defects in both the inhibition of hepatic glucose output and stimulation of glucose uptake by peripheral muscle tissues, resulting in increased gluconeogenesis and glycogen output to further elevate blood glucose levels (DeFronzo et al., 2015). Hyperglycemia can lead to insulin resistance through post-insulin receptor defects. This is mainly due to the inhibition of serine-threonine phosphorylation of insulin receptor and insulin receptor substrate-1 (IRS-1) and subsequent decrease in the activity of phosphatidylinositol-3 kinase (PI3K). This causes inhibition of glucose transporter 4 (GLUT4) translocation, inhibition of glucose uptake and phosphorylation, reduction of intracellular ATP level and reduction of glycogen synthesis (Grundy et al., 2004). Marsha et al. found that glucose induced insulin resistance through hexosamine biosynthesis pathway, and hyperglycemia increased the activity of hexosamine pathway in the cell that induced the formation of insulin resistance in muscle and adipose tissue (Marshall et al., 1991). Insulin non-sensitive tissue may be involved in diabetic vascular complications by promoting the synthesis of certain growth factors, such as transforming growth factor β (TGF-β) (Kolm-Litty et al., 1998). When blood sugar is too high and exceeds the renal glucose threshold, glucose, which cannot be metabolized, is excreted from the urine, leading to glucosuria (Dutchak et al., 2012).

Insulin secretion was affected by many factors. Our results of glucose tolerance test and insulin tolerance test showed that compared to the control group, OGTT of T2DM rats increased significantly in 30 min after glucose loading. Compared to the control C57BL/6J mice, the KK-Ay mice had significantly decreased reduction percentage of blood glucose in 40 min after administration of exogenous insulin. This indicates that the insulin secretion in the model animals was relatively insufficient and insulin resistance was present. The KK-Ay mice also had high FBG. In addition to the increase of FBG, KK-Ay also demonstrated insulin resistance characteristics. Moreover, urinary protein concentration and serum creatinine concentration in KK-Ay mice increased significantly. The pathological results also showed that the renal tissue of KK-Ay mice had mild or moderate level of glomerular mesangial matrix expansion, vacuolar degeneration of renal tubular epithelial cells and basophilic degeneration by week 4. Notably, our gene chip results showed that the PPAR signal pathway associated molecules Cyp4a1, Angptl4, Hmgcs2 were among the differentially expressed genes in both T2DM SD rats and KK-Ay mice.

In summary, the KK-Ay mice and T2DM SD rats not only have the characteristics of high FBG, but also have obvious characteristics of insulin resistance. The mice also showed the characteristics and progression of renal lesions. Together, these suggest that insulin resistance may be a key factor in the development of diabetic nephropathy but not the initiator.

### Metabolic Disorders of Lipids Play a Synergistic Role in the Pathogenesis and Progression of Diabetic Nephropathy

In diabetic patients, glucose metabolic disorder is often accompanied by lipid metabolic disorders, which lead to dyslipidemia, obesity, cardiovascular and cerebrovascular diseases. Excessive glucose and fat can induce insulin resistance and destroy the function of islet cells. Hyperlipidemia is the pathophysiological basis of insulin resistance which can not only exacerbates hyperglycemia but also causes a series of vascular complications in diabetes (Hirano, 2018). The increase in blood lipid concentration leads to increased blood viscosity, slow blood rheology and decreased blood flow, so that fat stores in the kidney and other non-adipose tissues lead to glomerulosclerosis (Chen et al., 2005). The increase of blood lipid may also change the structure of the fatty acid in the kidney and increase the pressure in the glomerular capillaries. Hyperlipidemia reduces fibrinolytic activity, resulting in vascular embolization of glomerular capillaries (Wang et al., 2005). The lipid metabolism of T2DM SD rats only appeared transiently abnormal at the 8th week. The levels of TG, TC, and LDL-C in the serum of KK-Ay mice increased significantly by the 4th week, which were accompanied by proteinuria. The gene microarray results showed that the key genes in the fatty acid synthesis and decomposition pathway such as *Acaa2, Ehhadh, Echs1* were down-regulated in KK-Ay mice, which was also consistent with the above hypothesis.

In summary, the KK-Ay mice developed hyperlipidemia and manifested elevated FBG. As the pathophysiological basis of insulin resistance, hyperlipidemia can aggravate insulin resistance. For the T2DM SD rats with transiently abnormal lipid metabolism, the dysfunction of lipid metabolism did not play an important role in the formation of kidney lesions, as the related parameters seemed to be recovered to the normal ranges as the controls during the later period. Therefore, we hypothesized that hyperlipidemia caused by abnormal lipid metabolism has a synergistic effect on the pathogenesis and progression of DN.

### Hemodynamic Changes Are One of the Important Pathogenesis of Diabetic Nephropathy

Abnormal renal hemodynamics is an important feature of the early stage diabetic nephropathy. Increased glomerular filtration rate plays a key role in the formation of diabetic nephropathy. This is primarily due to the increase in blood flow in each renal unit. The increase in the concentrations of blood glucose and lipid, the increase of blood viscosity, the slow blood rheology and the decrease of renal blood flow in diabetic patients lead to changes in renal hemodynamics and renal lesions aggravation (Wolf and Ziyadeh, 2007). A large number of studies have shown that renin-angiotensin system (RAS) is considered to be the main cause of renal injury and renal hemodynamic changes (Wada et al., 2011). Therefore, angiotensin convertase inhibitors are often used in the clinical treatment of renal diseases (Fernandez Juarez et al., 2013). The results of gene microarray showed that the expression of angiotensin II (Ang II), angiotensin convertase (ACE) and other factors in the two diabetic models were significantly increased. High glucose concentration can result in the increase of local Ang II in the kidney, which causes increased pressure in the glomerulus, the increase of renal glomerular filtration rate, and eventual glomerular damage (Lo et al., 2012). Ang II can also directly phosphorylate Smad protein by stimulating the expression of TGF-β in kidney and up-regulating the TGF-β receptor (Reudelhuber, 2010). ACE-2 is a key enzyme in the regulation and metabolism of angiotensin II (Ang 1-7) in RAS. It is also considered as an endogenous renal protective enzyme, which can effectively resist early onset proteinuria and alleviate glomerular pathological damage and renal fibrosis (Mori et al., 2014; Patel et al., 2014).

The following limitations of our study should be recognized. Firstly, we only observe the differences of renal lesions in two commonly used animal models of type 2 diabetes, but not included db/db mice, which are one of the most widely, used animal models of type 2 diabetic nephropathy for financial reasons. Secondly, the animal sample size is not large enough. Consider for its exploratory research, in the design of this experiment, there are only 54 type 2 diabetic rats and 18 kk-Ay mice were used as the starting point. At each time point, all the animals survived were tested for various indicators, and then three animals were randomly selected from each group for kidney sampling and subsequent pathology and sequencing. This can ensure the number of blood biochemical and urine biochemical indicators for each animal model, but also meet the requirements of three parallel samples for routine pathological and sequencing tests. Thirdly, the daily feed intake of animals was not measured, but body weight indirectly reflected the feed intake partly. Finally, animal species in this study. The bibliography supports that wistar rats maybe a better model for STZ-induced diabetes, while we selected the Sprague Dawley rats for my study, the main objective of this study was to compare the characteristics of DN between animal models of spontaneous type 2 diabetes and animals with chemically induced type 2 diabetes, in the future, we can consider to carry out in-depth research on the differences of diabetic nephropathy between SD rats and Wistar rats, specially for early kidney damage. Using mice with a specific mutation as a model for DM and DM related nephropathy, which is also one of the limitations of this study that needs to be focused on.

## Conclusion

Our study, for the first time, showed that KK-Ay mice with both high fasting glucose and insulin resistance, that had earlier onset with more uniform onset time and pathological characteristics compared with T2DM rats induced by low-dose STZ combining with high-fat-diet. The T2DM SD rats induced by low-dose STZ combining with high-fat-diet should not be used as a preclinical model for diabetic nephropathy because of the late onset time and unclear DN disease characteristics. Our systemic and comprehensive investigations on the occurrence and development of DN induced in these two common T2DM animal models, as well as our preliminary exploring on the underlying molecular mechanisms, provide significant guidance on the development of anti-DN drugs and valuable insight into the pathogenesis of DN in T2DM.

## Data Availability Statement

All datasets generated for this study are included in the article/Supplementary Material.

## Ethics Statement

The animal study was reviewed and approved by University Committee on the Use and Care of Animals (UCUCA) at the Beijing Yizhuang Biomedical Park Animal Center.

## Author Contributions

YL designed the experiments. YfL and HH performed the experiments and wrote the manuscript. YfL, HH, and RG analyzed the data. All authors edited the manuscript and gave final approval for the final version to be published.

## Conflict of Interest

HH was employed by the company Beijing Duan-Dian Pharmaceutical Research & Development Co., Ltd., Beijing, China. The remaining authors declare that the research was conducted in the absence of any commercial or financial relationships that could be construed as a potential conflict of interest.

## Abbreviations

AUC: Area Under the Curve
DM: Diabetes Mellitus
DN: Diabetic Nephropathy
FBG: Fasting Blood Glucose
ITT: Insulin Tolerance Test
LDL-C: Low-Density Lipoprotein-Cholesterol
OGCT: Oral Glucose Challenge Test
STZ: Streptozotocin
T2DM: Type 2 Diabetes Mellitus
TC: Total Cholesterol
TG: Triglyceride
UAE: Urinary Albumin Exclusion rate.
