# Supplementary material for: Dynamic Phenotypes and Molecular Mechanisms to Understand the Pathogenesis of Diabetic Nephropathy in Two Widely Used Animal Models of Type 2 Diabetes Mellitus
Source: Front Cell Dev Biol. 2020 Mar 19;8:172. doi: 10.3389/fcell.2020.00172 (PMC7098383; doi:10.3389/fcell.2020.00172)
Supplement: Supplementary file 1 [file Table_1.docx]

Supplementary Table 1 Number of animals at different time points

|  | 4 week | | 8 week | | 12 week | | 16 week | | 20 week | |
| --- | --- | --- | --- | --- | --- | --- | --- | --- | --- | --- |
| STZ-SD Rats | Biochemical indicators test | pathological  test | Biochemical indicators test | pathological  test | Biochemical indicators test | pathological test  /sequencing | Biochemical indicators test | pathological  test | Biochemical indicators test | pathological  test |
|  | 54 rats | 3 rats | 51 rats | 3 rats | 48 rats | 3 rats | 45 rats | 3 rats | 40 rats | 3 rats |
| KKAy mice | 18 mice | 3 mice | 15 mice | 3 mice | 12 mice | 3 mice | 9 mice | 3 mice | 6 mice | 3 mice |
